# Comparative Distribution and *In Vitro* Activities of the Urotensin II-Related Peptides URP1 and URP2 in Zebrafish: Evidence for Their Colocalization in Spinal Cerebrospinal Fluid-Contacting Neurons

**DOI:** 10.1371/journal.pone.0119290

**Published:** 2015-03-17

**Authors:** Feng B. Quan, Christophe Dubessy, Sonya Galant, Natalia B. Kenigfest, Lydia Djenoune, Jérôme Leprince, Claire Wyart, Isabelle Lihrmann, Hervé Tostivint

**Affiliations:** 1 Evolution des Régulations Endocriniennes, UMR 7221 CNRS, and Muséum National d’Histoire Naturelle, Paris, France; 2 Inserm, U982, University of Rouen, Mont-Saint-Aignan, France; 3 Laboratory of Neuronal and Neuroendocrine Differentiation and Communication, Institute for Research and Innovation in Biomedicine (IRIB), University of Rouen, Mont-Saint-Aignan, France; 4 Normandy University, University of Rouen, Mont-Saint-Aignan, France; 5 Laboratoire de Neurobiologie et Développement, CNRS UPR 3294, Institut Alfred Fessard, Gif-sur-Yvette, France; 6 Laboratory of Evolution of Neuronal Interactions, Sechenov Institute of Evolutionary Physiology and Biochemistry, Russian Academy of Sciences, St. Petersburg, Russia; 7 Institut du Cerveau et de la Moelle épinière, ICM, Inserm U 1127, CNRS, UMR 7225, Sorbonne Universités, UPMC University Paris 06 UMR S 1127, Paris, France; Institut National de la Recherche Agronomique (INRA), FRANCE

## Abstract

Urotensin II (UII) is an evolutionarily conserved neuropeptide initially isolated from teleost fish on the basis of its smooth muscle-contracting activity. Subsequent studies have demonstrated the occurrence of several UII-related peptides (URPs), such that the UII family is now known to include four paralogue genes called *UII*, *URP*, *URP1* and *URP2*. These genes probably arose through the two rounds of whole genome duplication that occurred during early vertebrate evolution. *URP* has been identified both in tetrapods and teleosts. In contrast, *URP1* and *URP2* have only been observed in ray-finned and cartilaginous fishes, suggesting that both genes were lost in the tetrapod lineage. In the present study, the distribution of *urp1* mRNA compared to *urp2* mRNA is reported in the central nervous system of zebrafish. In the spinal cord, *urp1* and *urp2* mRNAs were mainly colocalized in the same cells. These cells were also shown to be GABAergic and express the gene encoding the polycystic kidney disease 2-like 1 (pkd2l1) channel, indicating that they likely correspond to cerebrospinal fluid-contacting neurons. In the hindbrain, *urp1*-expressing cells were found in the intermediate reticular formation and the glossopharyngeal-vagal motor nerve nuclei. We also showed that synthetic URP1 and URP2 were able to induce intracellular calcium mobilization in human UII receptor (*h*UT)-transfected CHO cells with similar potencies (pEC50=7.99 and 7.52, respectively) albeit at slightly lower potencies than human UII and mammalian URP (pEC50=9.44 and 8.61, respectively). The functional redundancy of URP1 and URP2 as well as the colocalization of their mRNAs in the spinal cord suggest the robustness of this peptidic system and its physiological importance in zebrafish.

## Introduction

Urotensin II (UII) is a cyclic neuropeptide which was first isolated from the teleost urophysis on the basis of its spasmogenic properties [1]. Subsequent studies have shown that UII occurs in all vertebrate classes [2–6] where it exerts various biological effects including regulation of behaviors, neuroendocrine activities and control of cardiovascular functions [7], [8]. UII-related peptide (URP) is a peptide structurally related to UII which was first identified from the rodent brain [9] then in birds [10], amphibians [11] and teleosts [12]. In mammals, UII and URP have been shown to exert their action through a single receptor called UT, a member of the G protein-coupled receptor superfamily [13–16].

In teleosts, as in chondrichthyans, UII mRNA and/or peptide has been mainly found in Dahlgren cells of the caudal neurosecretory system [5,6,17–20] but its presence has also been reported in several subdivisions of the brain [19]. In contrast, in tetrapods, the *UII* gene is primarily expressed in motoneurons of the brainstem and spinal cord [21,22]. For its part, *URP* mRNA is mainly located in motoneurons in both tetrapods [11,23,24] and teleosts (Quan et al., unpublished results). Note that in lampreys, UII was isolated from an extract of the whole brain [6] but its precise localization in the central nervous system is still unknown.

Recently, the occurrence of two additional members of the *UII* gene family has been reported in teleosts, called *URP1* and *URP2* [25–28]. It has been proposed that both genes, together with *UII* and *URP* arose through the two rounds of whole genome duplication that occurred during early vertebrate evolution [26]. In agreement with this scenario, the existence of *URP1* and/or *URP2* genes has recently been reported in the spotted gar (a non-teleost actinopterygian) and elephant shark (a chondrichthyan) [27,28]. In contrast, the lack of *URP1* and *URP2* genes in tetrapods is believed to result from their loss in this lineage specifically [26]. The primary structure of both URP1 and URP2 is exactly the same in all fish species investigated so far.

Up to now, the expression pattern of the *URP1* gene has only been studied in one species, the Japanese eel (*Anguilla japonica*) [25] while the *URP2* gene expression has solely been reported in zebrafish (*Brachydanio rerio*) [26]. RT-PCR revealed that in both species *urp1* and *urp2* genes are mainly expressed in the brainstem and spinal cord. In zebrafish, it has been shown by *in situ* hybridization (ISH) that the *urp2* mRNA occurs in cells located along the ventral edge of the fourth ventricle and the ependymal canal. It has been suggested that these cells may correspond to cerebrospinal fluid- in cells located along the ventral edge of the fourth ventricle contacting neurons (CSF-cNs) [26].

In the present study, we report the distribution of *urp1* mRNA in the central nervous system of zebrafish and compare it to that of *urp2* mRNA. We demonstrate that *urp1* and *urp2* are mainly colocalized in the same cells in the spinal cord but not in the hindbrain. In the spinal cord, we provide evidence that cells containing both *urp1* and *urp2* mRNA are GABAergic and express the gene encoding the polycystic kidney disease 2-like 1 (pkd2l1) channel, indicating that they likely correspond to CSF-cNs. In the hindbrain, we show that *urp1*-expressing cells are located in the intermediate reticular formation and the glossopharyngeal-vagal motor nerve nuclei. Finally, we show that synthetic URP1 and URP2 are able to induce intracellular calcium mobilization in human UT-transfected Chinese hamster ovary cells (CHO) cells.

## Materials and Methods

### Chemicals and reagents

L-Amino acid residues were purchased from Senn Chemicals (Dielsdorf, Switzerland). Preloaded polyethylene glycol-polystyrene resins (Fmoc-Val-PEG-PS and Fmoc-Asn(Trt)-PEG-PS) and *O*-benzotriazol-1-yl-*N*,*N*,*N’*,*N’*-tetramethyluronium hexafluorophosphate (HBTU) were from Life Technologies (Saint Aubin, France, France). Acetonitrile, and *N*-methylpyrrolidinone (NMP) were from Biosolve Chimie (Dieuze, France). Diisopropylethylamine (DIEA), piperidine, trifluoroacetic acid (TFA), thallium (III) trifluoroacetate (Tl(OCOCF_3_)_3_) and other reagents were from Sigma-Aldrich (Saint-Quentin Fallavier, France).

### Peptide synthesis

Human UII (*h*UII; H-Glu-Thr-Pro-Asp-Cys-Phe-Trp-Lys-Tyr-Cys-Val-OH), mammalian URP (*m*URP; H-Ala-Cys-Phe-Trp-Lys-Tyr-Cys-Val-OH), URP1 (H-Ala-Cys-Phe-Trp-Lys-Tyr-Cys-Val-Thr-Asn-OH) and URP2 (H-Val-Cys-Phe-Trp-Lys-Tyr-Cys-Ser-Gln-Asn-OH) were synthesized (0.1 mmol scale) on a Fmoc-Val-PEG-PS or a Fmoc-Asn(Trt)-PEG-PS resin using an Applied Biosystems model 433A automatic peptide synthesizer and the standard procedures, as previously described [29,30]. All Fmoc-amino acids (1 mmol, 10 eq.) were coupled by *in situ* activation with HBTU (1.25 mmol, 12.5 eq.) and DIEA (2.5 mmol, 25 eq.) in NMP. Reactive side-chains were protected as follows: Cys, acetamidomethyl (Acm) thioether; Asn and Gln, trityl (Trt) amide; Ser, Thr and Tyr, tert-butyl (tBu) ether; Lys and Trp, tert-butyloxycarbonyl (Boc) carbamate and, Asp and Glu, tert-butyl (OtBu) ether. After completion of the chain assembly, cyclization of UII and URPs was performed by Tl(OCOCF_3_)_3_ oxidation as previously described [31]. Peptides were deprotected and cleaved from the resin by TFA as previously described [30,32]. Crude peptides were purified by reversed-phase HPLC (RP-HPLC) on a Vydac 218TP1022 C_18_ column (2.2 x 25 cm; Grace Discovery Sciences Alltech, Templemars, France) using a linear gradient (10–50% over 50 min) of acetonitrile/TFA (99.9:0.1, v/v) at a flow rate of 10 ml/min. Peptides were analyzed by RP-HPLC on a Vydac 218TP54 C_18_ column (0.46 x 25 cm; Grace Discovery Sciences Alltech) using a linear gradient (10–60% over 25 min) of acetonitrile/TFA (99.9:0.1, v/v) at a flow rate of 1 ml/min. The purity of all peptides was higher than 99.9%. The peptides were characterized by MALDI-TOF mass spectrometry on a Voyager DE-PRO (Applera, Courtaboeuf, France) in the reflector mode with α-cyano-4-hydroxycinnamic acid as a matrix.

### Animals

All zebrafish (*B*. *rerio*) lines were maintained and raised under standard conditions of 10/14 hours light cycle and water was regulated at 28.5°C, 500 μS and pH = 7.4. Wild-type animals (AB and Tüpfel) were obtained from local suppliers. The nacre mutants (*microphthalmia-a*, *mitfa*) which were selected for the embryo transparency were provided by Dr Jean-Pierre Levraud (Institut Pasteur, Paris, France). All procedures were approved by the Institutional Ethics Committee Cuvier at the Museum National d’Histoire Naturelle (protocol # 68–020; approval period from December 21, 2012 through December 19, 2017). In accordance to these guidelines, all efforts were made to minimize the number of animals used and their suffering.

### Reverse transcription-polymerase chain reaction (RT-PCR)

The expression profiles of *urp1* and *urp2* genes in adult zebrafish were examined by RT-PCR using gene-specific primers of each transcript (see S1 Table for the primer sequences). Total RNA was extracted from various tissues, including brain, spinal cord, eyes, skin, muscle, heart, spleen, gill, gas bladder, intestine, liver, kidney, ovary, and testis, following the protocol provided with RNAble reagent (Eurobio, Les Ulis, France) and further purified by using RNAeasy Plus Mini kit (Qiagen, Courtabœuf, France). For each tissue, 242 ng of total RNA was reverse transcribed by using ImProm-II Reverse Transcription System (Promega, Charbonnières, France). PCR was carried out using a MyCycler thermal cycler (Bio-Rad, Marne la Coquette, France) for 25 cycles (denaturation: 95°C, 2 min; annealing: 56°C, 30 sec; extension: 72°C, 30 sec). The zebrafish *β-actin* gene was amplified as an internal control to verify the integrity of all cDNA samples (primer sequences are given in S1 Table). Negative controls were performed without cDNA template. All PCR products were electrophoresed in 1% agarose gel stained with SybrSafe (Invitrogen, Pontoise, France) then detected under UV light with the Gel Doc XR^+^ System (Bio-Rad).

### Synthesis of the riboprobes for *in situ* hybridization

To generate the *urp1* and *urp2* ISH probes, two PCR fragments of 476 and 356 base pairs, respectively, were amplified from zebrafish brain and spinal cord RACE-ready cDNA (see S1 Table for the primer sequences) then subcloned into pGEM-T easy (Promega). Digoxigenin- and fluorescein-labeled probes were synthesized from the pre-linearized plasmid using SP6 or T7 RNA polymerases with the RNA Labeling Kit (Roche Diagnostics, Mannheim, Germany), according to the manufacturer’s instructions. Other probes, namely *islet-1* (*isl1*), *somatostatin 1* (*ss1*) and *gad*_*67*_, were synthesized as previously described [33,34].

### Sample preparation for *in situ* hybridization

Zebrafish embryos were fixed with 4% paraformaldehyde (PFA) in 0.1 M phosphate buffer at 4°C overnight and rinsed in 0.1 M phosphate-buffered saline, 0.1% Tween-20 (PBST). Adult zebrafish were deeply anesthetized in 0.02% MS-222 (Sigma-Aldrich) and killed by decapitation. Dissected brains and spinal cords were fixed with PFA as described above for embryos. For whole-mount ISH, fixed embryos or adult tissues were stored in 100% methanol at -20°C until use. For ISH of sections, fixed tissues were cryoprotected in 15% then 30% sucrose/PBS and embedded in Tissue-Tek (Sakura, Netherlands). Frontal or para-sagittal sections of brains (10–20 μm) and spinal cords (8–16 μm) were cut at -20°C using a cryostat (CM 3050, Leica, Nanterre, France), collected on Superfrost Plus slides (O. Kindler, Freiburg, Germany), dried at room temperature for 24 h and stored at -80°C until use. Free-floating sections (40 μM) were sometimes used for spinal cord observations.

### *In situ* hybridization procedures

Only ISH using fluorescent probes is described here, whole-mount ISH and ISH being exhaustively described elsewhere [26]. Fixed samples (whole-mount embryos and adult brain and spinal cords) were treated with 2% H_2_O_2_ in 100% methanol for 20 min, rehydrated through a series of solutions with descending ethanol percentage (75, 50, 25% ethanol all in PBST, for 10 min each) and permeabilized by treatment with 10 μg/ml of proteinase K in PBST for 1–20 min, depending on the developmental stage. They were then post-fixed in 4% PFA for 20 min, washed four times in PBST for 5 min, incubated in 2mg/ml glycine for 30 min and washed again in PBST. Samples were prehybridized in hybridation buffer (50% formamide, 5X saline-sodium citrate (SSC), 50 μg/ml heparin, 0.5 mg/ml yeast RNA (Sigma-Aldrich), 0.1% Tween-20) for 1 h at 65°C. Hybridization was performed overnight at 65°C in the same buffer containing the heat-denaturated digoxigenin-labeled riboprobe. Embryos were washed in a descending washing buffer (50% formamide, 5X SSC, 0.1% Tween-20) series (75, 50, 25 and 0% washing buffer all in 2X SSC for 15 min each) at 65°C. They were rinsed twice in 0.05X SSC for 30 min at the same temperature, once in 50% 0.05X SSC/50% PBST for 5 min, then twice in PBST. Following the final wash, brain and spinal cord were included in 3% agarose and sectioned at 50 μm using a vibratome (VT1000S Leica).

Embryos or adult tissue sections were then pre-incubated in blocking buffer (1% blocking reagent (Roche Diagnostics) in 100 mM maleic acid pH 7.5, 150 mM NaCl 0.1% Tween-20) for 1 h. Probe detection was carried out as follows: 1) incubation with specific anti-digoxigenin- and/or anti-fluorescein- antibodies conjugated to peroxidase (Roche Diagnostics; dilution: 1:300), 2) incubation in 0.0015% H_2_O_2_ for 30 min with the suitable fluorochrome- conjugated tyramide, and 3) peroxidase inactivation, by 2% H_2_O_2_. Labeled probes were revealed as previously described [35] by a home-made FITC- (Fischer scientific, Illkirch, France), TAMRA- (Life Technologies) or Cy5- (Perkin Elmer, Courtaboeuf, France) conjugated tyramide (protocol available on Xenbase: http://www.xenbase.org/other/static/methods/FISH.jsp). After extensive washes in PBST (at least five times for 20 min each), samples were postmounted on Superfrost Plus slides (O. Kindler) in Mowiol (Sigma Aldrich). For double fluorescent ISH, the fluorescein-labeled probe was revealed first. The specificity of each probe was verified using the sense probe as negative control (data not shown).

### Combined fluorescent ISH and immunofluorescence

ISH was performed before IHC as described above and revealed using FITC-, TAMRA- and/or Cy5-conjugated tyramide. After several washes in 0.1% Triton/0.1 M PBS, tissues were pre-incubated in 4% BSA, 0.1% Triton, 0.1 M PBS for 1 h. Primary antibodies (rabbit anti-GAD_65/67_ (Abcam, Cambridge, UK) 1:100 or goat anti-choline acetyltransferase (ChAT, Millipore), 1:100) were incubated in the same solution overnight at 4°C. Secondary antibodies (donkey anti-rabbit IgG Alexa Fluo 488/596 (Invitrogen) 1:500, or donkey anti-goat IgG Alexa Fluor 488 (Invitrogen) 1:500) were incubated after three washes in 0.1% Triton/0.1 M PBS for 2 hours, then washed twice in PBS. The specificity of the immunostaining was verified by omitting the primary antibodies (data not shown).

### Image acquisition

Samples stained by BM Purple were imaged using a Leica DM 5500 B microscope connected to LAS V4.1 software. Samples stained by tyramide system amplification were imaged by the Olympus FV1000 or Zeiss LSM700 confocal microscopes using 405, 488, 543 and 633 nm laser lines. Images were processed using the ZEN (Zeiss, Marly Le Roi, France), Fiji [36] and Adobe Illustrator (Adobe Systems, Mountain View, CA, USA) softwares.

### Intracellular calcium mobilization assays

CHO cells stably transfected with the human UII receptor (*h*UT-CHO) (Chatenet *et al*. 2004) were plated at a density of 4 x 10^4^ cells/well in flat clear bottom black 96-well plates. Measures of intracellular calcium mobilization were performed as previously described (Dubessy *et al*. 2008) with slight modifications. Briefly, after 24 h in culture, cells were incubated for 1 h in a humidified incubator (37°C, 5% CO_2_) with 2 μM Fluo-4 acetoxymethyl ester (AM) calcium dye (Life Technologies) in Hank’s Buffer Saline Solution (HBSS; Life Technologies) buffered with 5 mM HEPES and supplemented with 2.5 mM probenecid (Sigma-Aldrich, Saint-Quentin Fallavier, France). Cells were washed twice with HBSS/HEPES/probenecid to remove Fluo-4 AM from the incubation medium and incubated in 150 μl of the same medium at 37°C for 15 min. Fluorescence was recorded using a Flexstation 3 fluorescence plate reader system (Molecular Devices, Saint-Grégoire, France) during 180 sec with an excitation wavelength of 485 nm, an emission wavelength of 525 nm and a cutoff filter of 515 nm. After 18 sec recording in basal conditions, 50 μl of graded concentration (10^-12^ to 10^-6^ M) of different peptides (4-fold final concentration) was added to the incubation medium with the built-in 8 channel pipettor at a rate of 47 μl/sec to assess their agonistic activity. After subtraction of mean fluorescence background from control wells without Fluo-4 AM, baseline was normalized to 100% and fluorescence peak values were determined for each concentration of peptide. Potency (EC_50_) and efficacy (E_max_) were calculated with the Prism 5.0 software (GraphPad Software In., La Jolla, CA, USA) using a four-parameter logistic equation to fit peak fluorescence data.

### Statistical analysis

For intracellular calcium mobilization assays, results from 7–13 independent experiments were expressed as mean ± SEM of Log(EC_50_) and plotted with box and whiskers. The normality of each data set was verified with the Shapiro-Wilk’s test and a one-way ANOVA test followed by a Tukey’s multiple comparison test to compare Log(EC_50_) between each peptide. Differences were considered significant where *P<0*.*05*.

## Results

### Both *urp1* and *urp2* genes are expressed in the hindbrain and spinal cord in embryo and adult zebrafish

**Fig 1:**
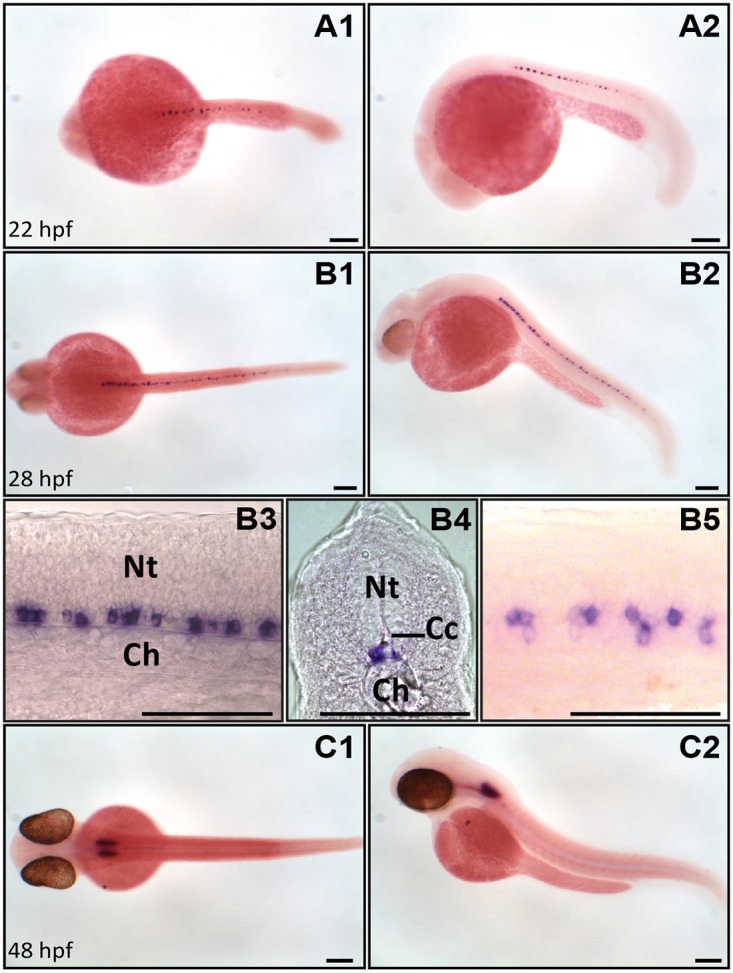
*urp1* mRNA is restricted to the ventral spinal cord and hindbrain at early stages of development in zebrafish. Expression of *urp1* revealed by ISH (BM purple, violet) on nacre embryos at 22 **(A)**, 28 **(B)** and 48 hpf **(C)**. At 22 hpf and 28 hpf, *urp1* ^+^ cells occur only in the spinal cord at the level of the lateral floor plate **(A, B)**, while from 48 hpf, they are mainly visible in the hindbrain **(C)**. **A1**, **B1**, **B5** and **C1**, dorsal views; **A2**, **B2, B3** and **C2**, lateral views with dorsal up; **B4**, coronal section with dorsal up; all embryos oriented anterior left; **B3** and **B5** are details at higher magnifications of B2 and B1, respectively. Ch, chord; Cc, central canal; Nt, neural tube; Scale bar: 100 μm.

The expression of the *urp1* gene in zebrafish embryos was investigated by ISH at stages 18, 20, 22, 28, 30, 36 and 48 hours post fertilization (hpf) stages. The first *urp1*-expressing (*urp1* ^+^) cells were detected at 22 hpf in the rostral half of the spinal cord (Fig. 1A). From 22 to 36 hpf, the *urp1* staining expanded more caudally within the spinal cord (Fig. 1B1, 2). At any stage analyzed, *urp1* ^+^ cells were exclusively located at the base of the neural tube, ventral to the central canal (Fig. 1B3,4). These cells, which were distributed in two rows along the midline, belong to the lateral floor plate (Fig. 1B4,5). At 48 hpf, the *urp1* mRNA was primarily detected in a tight bilateral cluster of cells in the hindbrain, while it was hardly detectable in the spinal cord (Fig. 1C).

**Fig 2:**
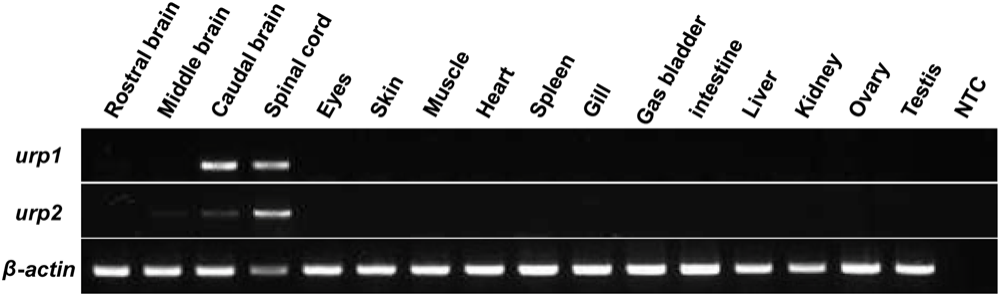
*urp1* and *urp2* mRNAs are exclusively detected in the brain and spinal cord in adult zebrafish. Tissue distribution of *urp1* and *urp2* mRNAs assessed by RT-PCR. Parallel amplification of zebrafish β-actin mRNA served as internal control. NTC, non-template control.

RT-PCR was used to determine the distribution of *urp1* and *urp2* mRNAs in different organs of adult zebrafish. Both mRNAs were detected in the hindbrain and the spinal cord (Fig. 2). The *urp2* mRNA was also found in the middle part of the brain, as previously reported [26]. In all the others tissues tested, the expression of *urp1* and *urp2* genes was undetectable (Fig. 2).

**Fig 3:**
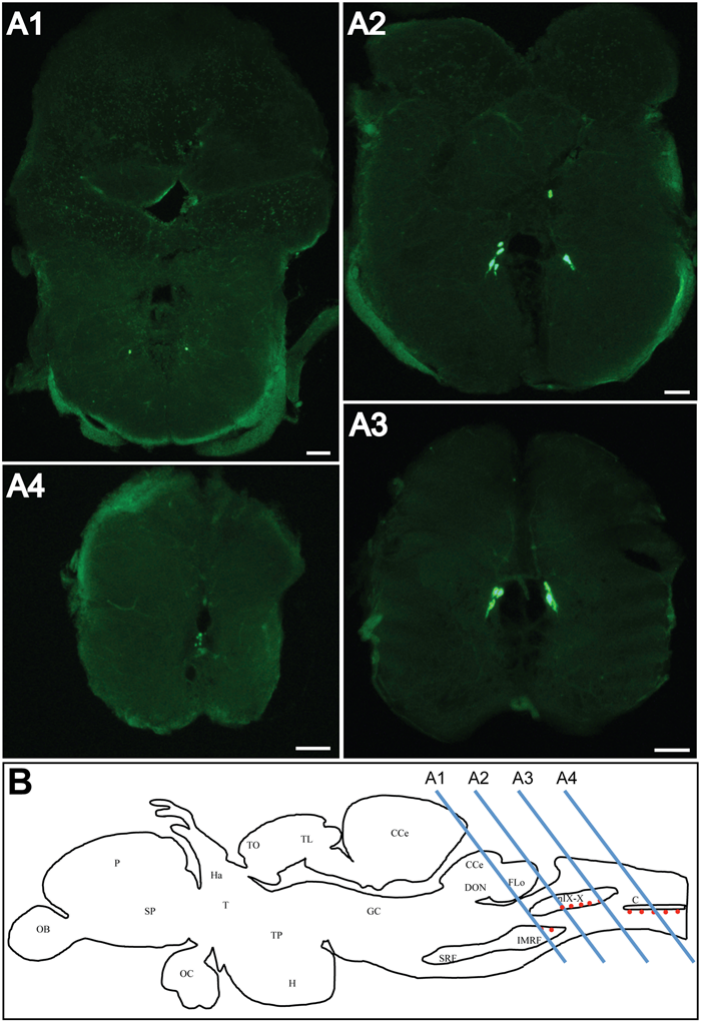
*urp1* mRNA is found in the caudal part of the hindbrain in adult zebrafish. Expression of *urp1* revealed by fluorescent ISH (FITC, green) on coronal sections of adult brain **(A)**. *urp1* mRNA is visible in neurons located in the intermediate reticular formation **(A1**) and the region of the glossopharyngeal-vagal motor nerve nuclei (**A2–A3**). More caudally, at the level of the junction between hindbrain and spinal cord, *urp1* mRNA occurs at the ventral edge of the central canal **(A4)**. Schematic sagittal view of an adult zebrafish brain depicting the distribution of *urp1* mRNA (red dots). Levels of sections shown in A are indicated. The anatomical structures are designated according to [38] **(B)**. CC, cerebellar crest; C, central canal; CCe, corpus cerebelli; DON, dorsal octavolateralis nucleus; EW, Edinger-Westphal nucleus; FLo, facial lobe; Ha, habenula; H, hypothalamus; IMRF, intermediate reticular formation; MO, medulla oblongata; NC, commissural nucleus of Cajal; nIX-X, glossopharyngeal-vagal motor nerve nuclei; OB, olfactive bulbs; OC, optic chiasma; P, pallium; PN, preopic nucleus; RV, rhombencephalic ventricle; SCsm, spinal cord somatomotor neurons; SP, subpallium; T, thalamus; TO, tectum opticum; TL, torus longitudinalis; TP, posterior tuberculum; TS, torus semicircularis; VLo, vagal lobe. Scale bars: 100 μm.

In line with PCR results, ISH analysis showed the presence of *urp1* ^+^ cells in the medulla oblongata (Fig. 3A, B) at the level of the intermediate reticular formation (Fig. 3A1) and the roots of the glossopharyngeal and vagal nerves (Fig. 3A2,3) [37,38]. Most caudal, a group of *urp1* ^+^ cells was found at the junction between the rhombencephalon and spinal cord, at the ventral edge of the central canal (Fig. 3A4).

**Fig 4:**
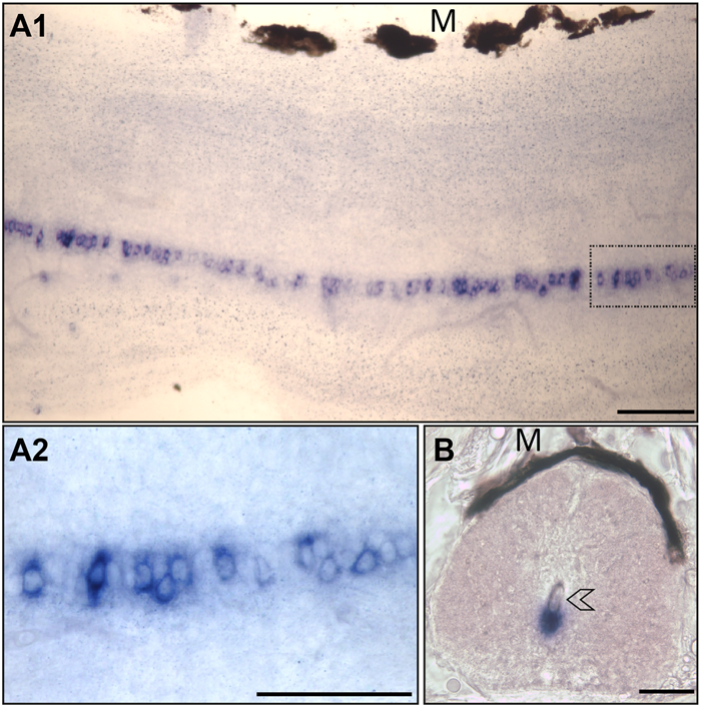
*urp1* mRNA occurs in cells located along the ventral edge of the central canal of spinal cord in adult zebrafish. Expression of *urp1* revealed by ISH (BM purple, violet) on free-floating sections of adult spinal cord. *urp1* ^+^ cells form a quasi-continuous line at the ventral edge of the central canal **(A)**. *urp1* ^+^ cells are in close contact to the lumen of the central canal (arrowhead) **(B)**. **A1** and **A2**, lateral sections with dorsal up; **B**, coronal section with dorsal up. *urp1* ^+^ cells boxed in **A1** are shown in **A2** at higher magnification. M, melanocytes. Scale bars: 50 μm.

In the spinal cord, *urp1* ^+^ cells formed a quasi-continuous column in the ventral margin along the central canal (Fig. 4A1,2) with exception to its more caudal part (data not shown). In this regard, it is noteworthy that no labeling was observed in Dahlgren cells nor in the urophysis. As shown in Fig. 4B, *urp1* staining occurred in close contact to the lumen of the central canal. The distribution of the *urp2* mRNA has been previously reported both in the midbrain and the spinal cord [20]. S1 Fig. highlights the occurrence of *urp2* ^+^ cells at the ventral edge of the fourth ventricle.

### *urp1* and *urp2* mRNAs are mainly localized in the same cells of the spinal cord

**Fig 5:**
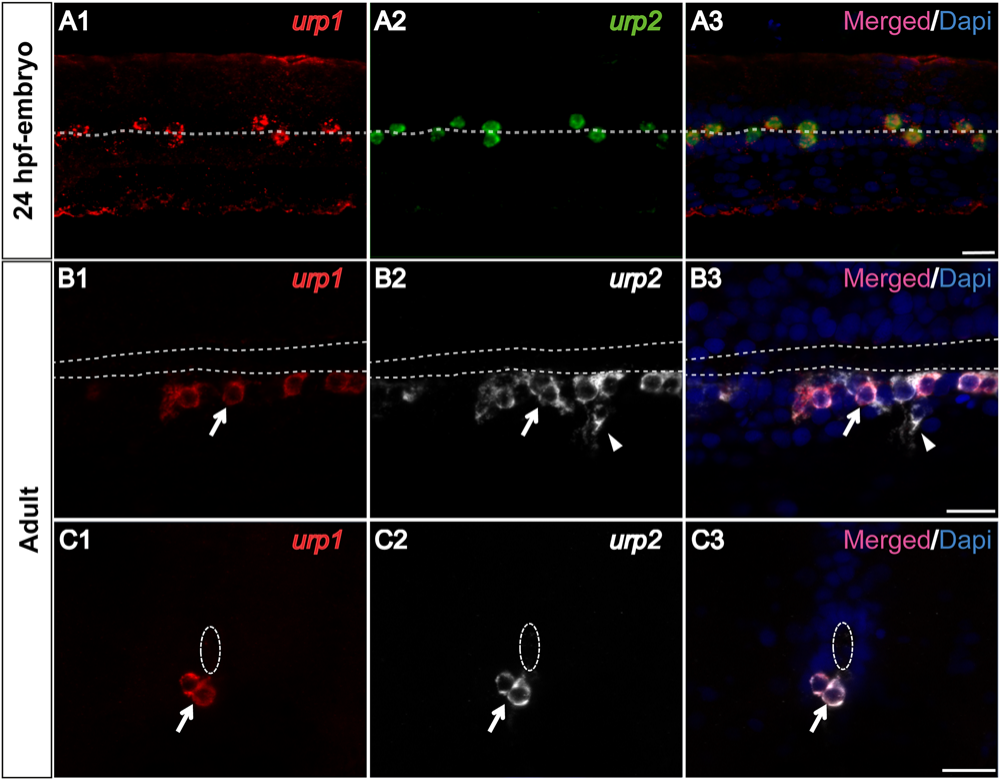
*urp1* and *urp2* mRNAs are mainly coexpressed in the same spinal cord cells in zebrafish. Simultaneous expression of *urp1* and *urp2* revealed by double fluorescent ISH (TAMRA, red for *urp1*, FITC, green for *urp2* and DAPI in blue) on 24 hpf-embryo **(A)** and adult spinal cord sections **(B, C)**. In embryo, all the stained cells contain both *urp1* and *urp2* mRNAs **(A)**. In adult, although most of the stained cells are doubly-positive for *urp1* and *urp2* (arrow), some of the *urp2* ^+^ cells are devoid of any *urp1* mRNA (arrowhead). The white dash line indicates the central canal. **A**, dorsal view; **B**, sagittal section with dorsal up; **C**, coronal section with dorsal up. Scale bars: 15 μm.

The expression pattern of the *urp1* gene in the spinal cord appeared very similar to that of the *urp2* gene [26]. To assess whether the two genes are expressed in the same cells, we performed double fluorescent ISH using *urp1* and *urp2* antisense probes, both in embryos and adults. In 24 hpf-embryos, we observed that all stained cells contained both *urp1* and *urp2* mRNAs (Fig. 5A). In adults, all *urp1* ^+^ cells were stained by the *urp2* probe (Fig. 5B,C), while about 20% of the *urp2* ^+^ cells did not contain the *urp1* mRNA (Fig. 5B).

### Cells containing both *urp1* and *urp2* mRNA in the spinal cord also express markers of the CSF-contacting neurons

**Fig 6:**
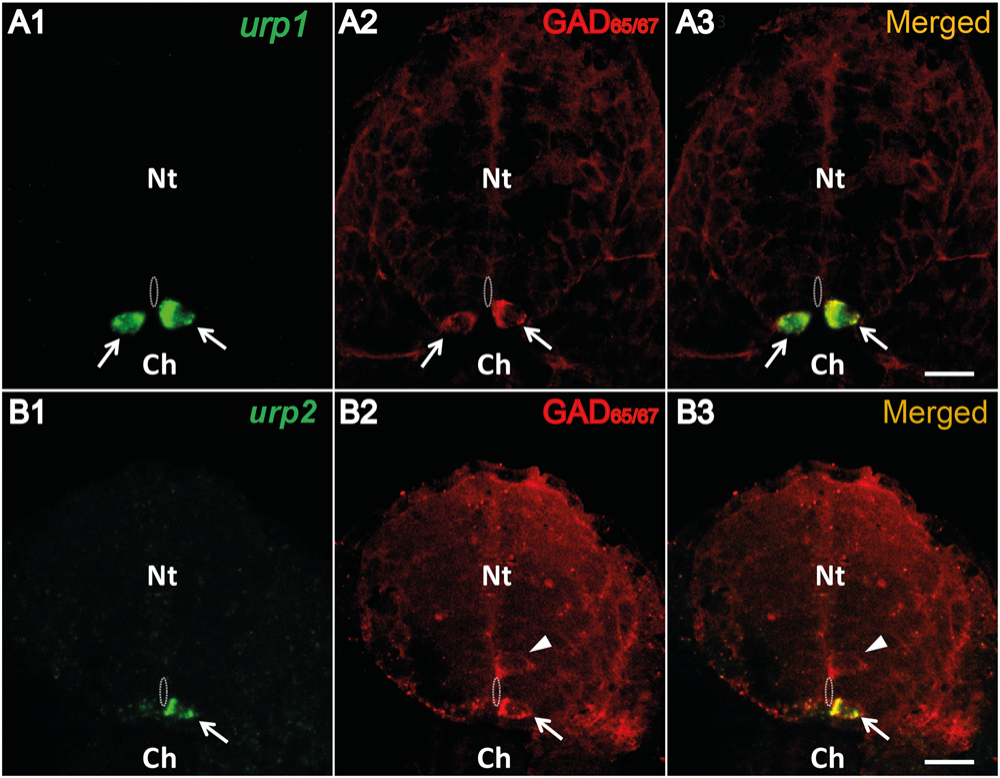
Both *urp1* ^+^ and *urp2* ^+^ cells are GABAergic neurons in the zebrafish embryo. *urp1* **(A)** and *urp2* **(B)** expression revealed by fluorescent ISH (FITC, green) on 24 hpf-embryo, together with a fluorescent immunostaining for GAD_65/67_ (Alexa Fluor 546, red). Both *urp1* ^+^ and *urp2* ^+^ cells are GAD^+^ (arrows). Note that only ventral KA (KA”) cells are doubly stained. In contrast, dorsal KA (KA’) cells are GAD^+^ but do not express *urp1* (arrowhead). The white dash line indicates the central canal. **A** and **B**, coronal sections with dorsal up. Scale bars: 15 μm.

*urp1* ^+^ cells, as *urp2* ^+^ cells, were located in close contact to the central canal, indicating that they may both correspond to CSF-cNs. In zebrafish embryos, it has been shown that spinal CSF-cNs are GABAergic [39,40]. Therefore, we asked whether *urp1* ^+^ and *urp2* ^+^cells may express GABAergic markers. In 24 hpf-embryos, single fluorescent ISH using either the *urp1* or *urp2* probe followed by IHC for GAD_65/67_ showed that all *urp1* ^+^ cells as well as all *urp2* ^+^ cells were GAD_65/67_ immunoreactive (Fig. 6A, B, arrows).

**Fig 7:**
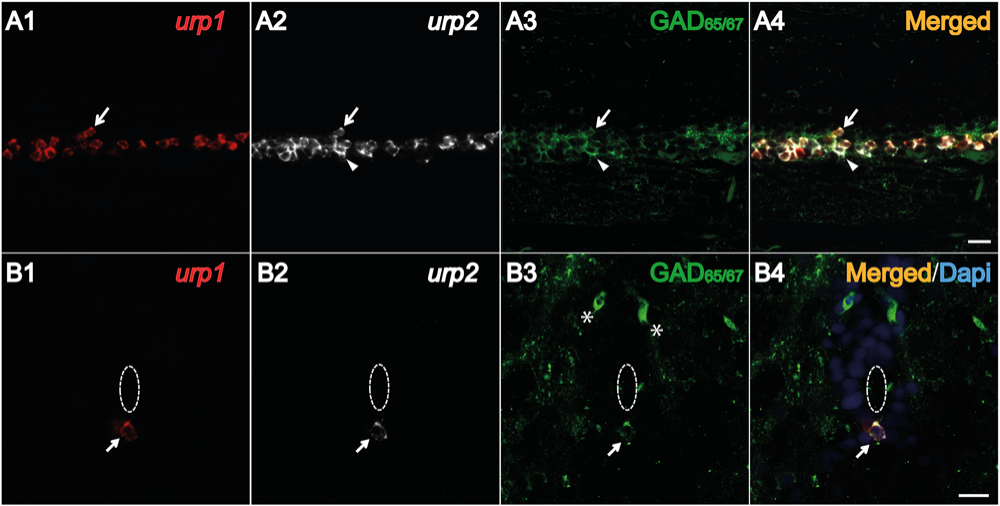
Both *urp1* ^+^ and *urp2* ^+^ cells in the spinal cord are GABAergic neurons in adult zebrafish. Simultaneous expression of *urp1* and *urp2* revealed by double fluorescent ISH (TAMRA, red for *urp1* and Cy5, white for *urp2*) in adult spinal cord, coupled to a fluorescent immunostaining for GAD_65/67_ (Alexa Fluor 488, green) **(A**, **B)**. Both *urp1* ^+^ and *urp2* ^+^ cells are GAD^+^ **(A, B)**. Arrows designate triple-stained cells **(A, B)**. Note the occurrence of some doubly-positive cells (*urp2*/GAD) that do not contain any *urp1* (arrowhead) **(A)**. Asterisks designate GABAergic interneurons located at the dorsal part of the spinal cord. The white dash line indicates the central canal. **A**, sagittal section with dorsal up; **B**, coronal section with dorsal up. Scale bars: 15 μm.

The same results were obtained in adults by triple staining experiments using both *urp1* and *urp2* probes combined with an anti-GAD_65/67_ antibody. All *urp1* ^+^ and *urp2* ^+^cells were GAD_65/67_ immunoreactive (Fig. 7A, B, arrows). As mentioned above, some of the *urp2* ^+^ cells did not contained the *urp1* mRNA (Fig. 7A4, arrowhead).

**Fig 8:**
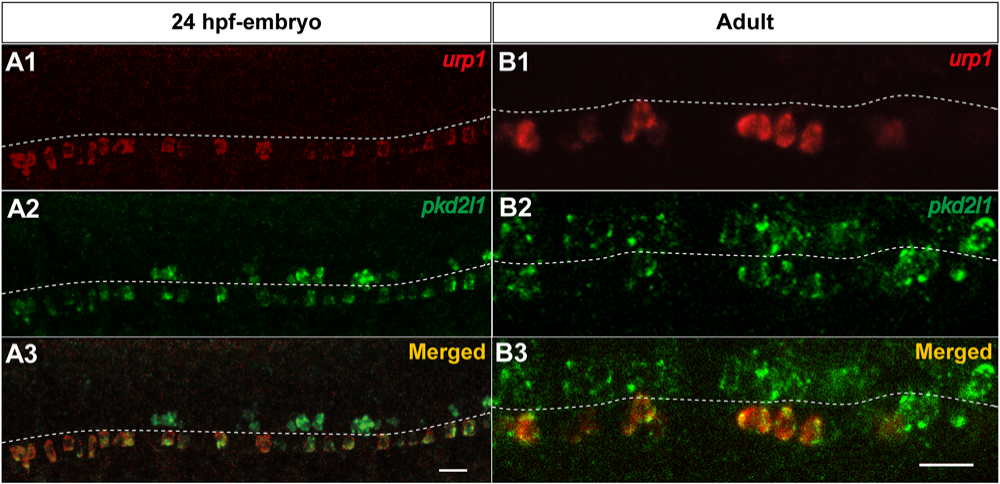
*urp1* ^+^ cells express *pkd2l1*, a specific marker of spinal cerebrospinal fluid- contacting neurons in zebrafish. Simultaneous expression of *urp1* and *pkd2l1* revealed by double fluorescent ISH (TAMRA, red for *urp1* and FITC, green for *pkd2l1*) on 24 hpf-embryo **(A)** and adult spinal cord sections **(B)**. *pkd2l1* mRNA is distributed in two rows of cells along the rostro-caudal axis of the spinal cord both in embryo and adult **(A2, B2)**. All the *urp1* ^+^ cells are *pkd2l1* ^+^ **(A1,3**, **B1,3)** but only the ventral *pkd2l1* ^+^ cells are *urp1* ^+^. The white dash line indicates the central canal. **A**, lateral views; **B**, sagittal sections with dorsal up. Scale bars: 20μm.

The gene encoding for the calcium-permeable PKD2L1 channel has been recently reported as a specific marker of CSF-cNs in various vertebrate species including zebrafish [41–43]. To determine whether *urp1* ^+^ cells express *pkd2l1*, we performed double fluorescent ISH using *urp1* and *pkd2l1* probes, both in embryos and adults. As depicted in Fig. 8A, in 24 hpf-embryos, all *urp1* ^+^ cells contained *pkd2l1* mRNA, but their localization was restricted to the ventral subpopulation of *pkd2l1* ^+^cells. The same results were observed in adults, since *urp1* ^+^ cells represented a fraction of *pkd2l1* ^+^cells located at the ventral edge of the central canal (Fig. 8B). Likewise, all *urp2* ^+^ cells were identified as *pkd2l1* ^+^ both in embryo and adult (data not shown).

### Hindbrain cells containing *urp1* mRNA also express motoneuron markers

**Fig 9:**
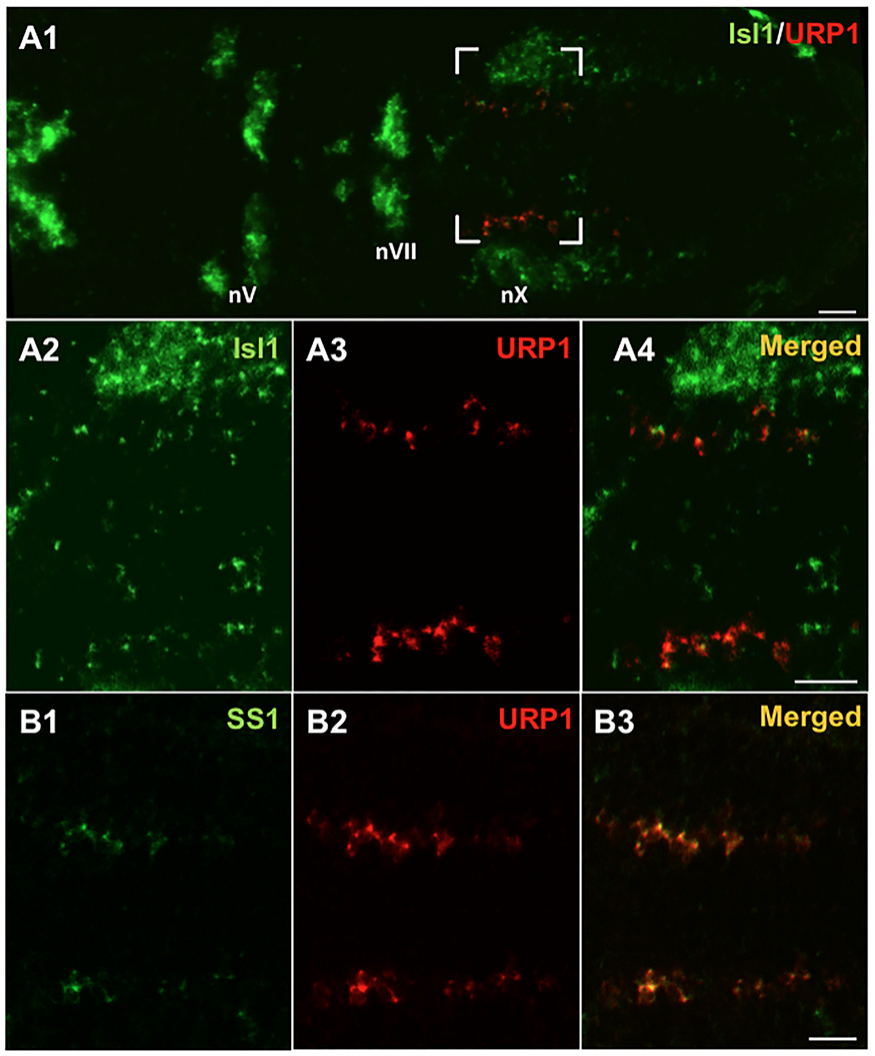
*urp1* ^+^ cells express *isl1* and *ss1*, two markers of the vagus motor nucleus in zebrafish embryo. Simultaneous expression of *urp1* and *isl1* **(A)** or *ss1* **(B)** revealed by double fluorescent ISH (TAMRA, red for *urp1* and FITC, green for *isl1* or *ss1*) on 48 hpf-embryo. *urp1* ^+^ cells are located at the level of the medial motor nucleus of the vagus. Most of them appear to be both *isl1* ^+^ and *ss1* ^+^. All pictures are dorsal views with anterior left. The boxed region in **A1** is shown at higher magnification in **A2–A4** and the same region is shown in **B**. nV, trigeminal nerve motor nuclei; nVII, facial nerve motor nuclei; nX, vagal nerve motor nuclei. Scale bars: 20 μm.

To better characterize the *urp1* gene expression pattern in the hindbrain, we tested the colocalization of *urp1* mRNA with different markers, namely *isl1*, *ss1*, ChAT and GAD. *isl1* is a member of the LIM/homeobox gene family which is expressed in all postmitotic motoneurons at early stages of development [44]. Its expression pattern is particularly suitable to discriminate the different types of cranial motor nuclei in the hindbrain. In the 48 hpf-embryo, double fluorescent ISH using *urp1* and *isl1* probes revealed that all *urp1* mRNA was exclusively present in *isl1* ^+^cells (Fig. 9A) at the level of the medial motor nucleus of the vagus [45]. Double fluorescent ISH using the *urp1* and *ss1* probes was also carried out, since *ss1* was previously shown to be expressed in neurons of the vagal motor nucleus [33]. As depicted in Fig. 9B, all *urp1* mRNA colocalized with *ss1* mRNA. It is noteworthy that *urp1* ^+^ cells were detected only in the most ventral part of the *ss1*-positive area (data not shown).

**Fig 10:**
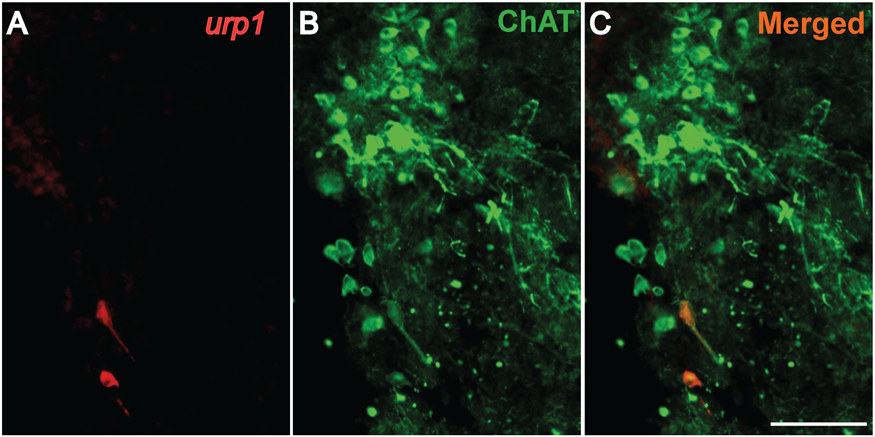
*urp1* ^+^ cells in the hindbrain are cholinergic neurons expressing ChAT in adult zebrafish. *urp1* expression revealed by fluorescent ISH (TAMRA, red) on coronal sections of adult brain, together with a fluorescent immunostaining for ChAT (Alexa 488, green). *urp1* ^+^ cells express ChAT. Scale bars: 100 μm.

To characterize the *urp1* ^+^ cells in the adult hindbrain, we performed single fluorescent ISH using the *urp1* probe followed by IHC with ChAT, a marker of cholinergic neurons [38,46]. As shown in Fig. 10, *urp1* ^+^ cells located in the glossopharyngeal-vagal motor nerve nuclei were weakly stained by the anti-ChAT antibody. Double fluorescent ISH using *urp1* and *gad*_*67*_ probes did not reveal any double-labeled cells (S2 Fig.), in contrast to what was observed in the spinal cord. Note that in the intermediate reticular formation, *urp1* ^+^ cells were both ChAT- and *gad*_*67*_-negative (data not shown).

### URP1 and URP2 are equipotent to activate the *h*UT

**Fig 11:**
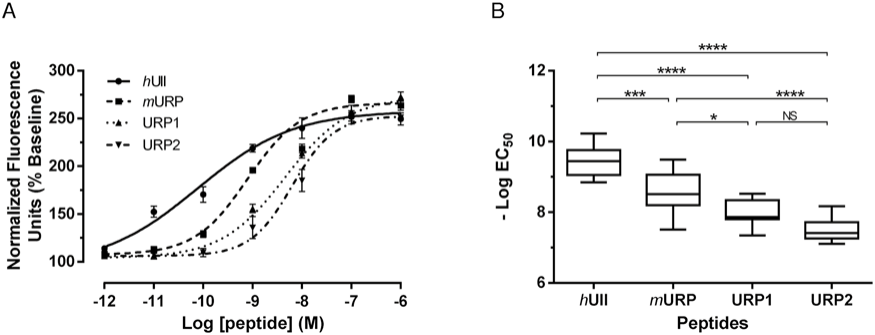
URP1 and URP2 are equipotent to induce intracellular calcium mobilization in a *h*UT-transfected CHO cell line. Representative dose-response curves of *h*UII (●), *m*URP (■), URP1 (▲) and URP2 (▼) on the intracellular calcium mobilization **(A)**. The values are expressed as percentages of the baseline and each point is the mean (± S.E.M.) of 3 replicates. Experimental data were fitted using a four-parameter logistic equation. The potencies of 7–13 independent experiments for each peptides were plotted as—Log(EC_50_) with box and whiskers **(B)**. Values were considered as statistically different as assessed by analysis of variance followed by Tukey’s post-test, n.s., not significant, *p < 0.05, **p < 0.01, ***p < 0.001, ****p < 0.0001.

As a step to elucidate the physiological actions of URP1 and URP2, we tested the *in vitro* activities of both peptides using transfected CHO cells expressing the human UT using a calcium mobilization assay. Synthetic URP1 and URP2 induced a robust increase in intracellular calcium in *h*UT-CHO cells with similar efficacies (around 200–250%) than those evoked by *h*UII and *m*URP (Fig. 11A). Of note, URP1 and URP2 were equipotent to activate the *h*UT (pEC_50_ = 7.99 ± 0.15 and 7.52 ± 0.11 respectively). Nevertheless, they were respectively 28 and 83 times less potent than *h*UII (pEC_50_ = 9.44 ± 0.12) to mobilize intracellular calcium. *m*URP, which is also the natural ligand of *h*UT, exhibited an intermediate potency (pEC_50_ = 8.61 ± 0.16) statistically distinct from the others tested peptides (Fig. 11B).

## Discussion

The aim of the present work was to compare the distributions and *in vitro* activities of the urotensin II-related peptides URP1 and URP2 in zebrafish. Previous studies had shown that *urp2* gene expression occurs in cells contacting the central canal in the spinal cord [26], while *urp1* mRNA was confined in the caudal neurosecretory system [25]. Our data do not confirm the presence of *urp1* in the caudal spinal cord reported in the eel [25]. Instead *urp1* ^+^ cells were found, as *urp2* ^+^ cells, close to the central canal of the spinal cord. However, in contrast to *urp2* mRNA which exhibits alternate zones of strong and weak staining [26], *urp2* mRNA was present approximately at the same intensity along the entire length of the spinal cord. Worth mentioning, all *urp1* ^+^ cells were *urp2* ^+^, while the opposite was not necessarily true, at least in adults.

On the basis of a former study reporting the occurrence of UII-like immunoreactive material in CSF-cNs [47], we suggested that *urp2* ^+^cells might be CSF-cNs [26]. CSF-cNs are neurons, described in all vertebrate groups [48–50], located on the edge of the neural tube lumen, so-called because they are in direct contact with the CSF via their dendritic pole. In *Xenopus* as in zebrafish, CSF-cNs have been shown to project an ascending axon ventrally in the spinal cord [39,51–53]. Moreover, analysis of the UII-like immunoreactive-containing system from various fish species showed that CSF-cNs send fibers to several brain regions, including medulla oblongata, thalamus, hypothalamus and telencephalon, but also in the spinal cord ventrolateral surface [47,54].

Although their functions remain largely unknown, CSF-cNs are classically considered as sensory neurons, but the exact nature of their stimuli is not clearly established. Recent studies have shown that they are GABAergic (see [42] for review). They also express PKD2L1, a transient receptor potential channel that could play a role as a sensory receptor [42,43], in good agreement with studies describing these neurons as chemoreceptors or mechanoreceptors sensing the CSF chemical composition and/or movements [42,50]. In addition, using the powerful optogenetic approach, Wyart et al. [53] have shown that CSF-cNs might be involved in motor control as part of the neuronal network that controls spontaneous swimming movement of zebrafish larvae. Thus, our data demonstrate that *urp1* ^+^ and *urp2* ^+^ cells both express GABA and *pkd2l1* indicating that they actually are CSF-cNs and that URP1 and URP2 might have a role in the neuronal network that controls spontaneous swimming in zebrafish, at least in larvae.

It is likely that the presence of URP1 and/or URP2 in CSF-cNs is an ancestral feature of gnathostomes since the occurrence of CSF-cNs containing UII-like immunoreactivity has also been reported in a chondrichthyan species, *Hydolagus collieri* [54]. Beside URP1 and URP2, several other neuropeptides have been detected in CSF-cNs, such as vasoactive intestinal peptide (VIP) and SS [53,55–57]. In this respect, it is noteworthy that in coho salmon *Oncorhynchus kisutch*, SS- and UII-immunoreactive material do not localize in the same CSF-cNs [58] suggesting the occurrence of distinct CSF-cN types. Indeed, in zebrafish embryos, in which GABAergic CSF-cNs were named Kolmer-Agduhr (KA) cells to distinguish them from ciliated ependymal cells [51], KA cells have been divided into two subpopulations, namely KA’ and KA” cells, on the basis of their developmental origin and location. Whereas the dorsal KA’ cells are derived from *olig2* ^+^ motoneuron precursors, the more ventral KA” cells develop from the lateral floor plate [34,42,59]. Our results showing that *urp1* and *urp2* gene expression was restricted to the KA” ventral subpopulation, reinforce the idea of the CSF-cNs diversity and reveal *urp1* and *urp2* as new markers for KA” cells in zebrafish. In adults, the fact that *urp1* ^+^ and *urp2* ^+^cells also occur in a ventral position indicates that KA” cells conserve their location relative to the central canal during development. Whether all these different neuropeptides could help define additional types of CSF-cNs remains to be determined.

At the brain level, *urp1* ^+^ cells were restricted to the rhombencephalon. In embryos, they were observed from 48 hpf, *i*.*e*. more than 24 h later than those detected in the spinal cord, in a small bilateral area also expressing *isl1* and *ss1* genes. In agreement with previous studies [33–44], these cells likely correspond to motoneurons in the vagal motor nucleus. Supporting this idea, most of the *urp1* ^+^ cells in adults were identified as cholinergic neurons located in the same nucleus [38]. These data indicate that *urp1* gene expression in the vagal motor nucleus persists throughout the entire development period until adulthood. It is noteworthy that UII and URP are known to be expressed mainly in motoneurons in tetrapods [11,21–24,60]. While *urp1* and *urp2* gene expression largely overlap in the spinal cord, their respective patterns differ completely in the brain since *urp2* ^+^ cells appear to form an extension of the spinal *urp2* CSF-cNs system below the fourth ventricle [26].

In trout (*O*. *mykiss*) and eel (*A*. *japonica*), central injection of UII evokes an increase in arterial blood pressure and heart rate [25,62–65]. Similar effects have been reported with URP1 [25]. It has been suggested that UII and URP1 may act directly at the central level, even though their site of action are still uncertain [25]. The present data show that the glossopharyngeal-vagal motor nerve nuclei are a putative source of URP1 indicating that URP1, rather than UII absent here [17,19,20,25], may act as a central regulator of the cardiovascular activity. Likewise, central pharmacological effects of UII on motor activity [61] could be achieved by URP1 expressed in the intermediate reticular formation, one type of zebrafish hindbrain nuclei projecting to the spinal cord [65].

To date, the precise mechanism of the URP1 action is unkown. Calcium mobilization assay showed here that synthetic URP1 and URP2 are active peptides toward human urotensin II receptor. This is consistent with the presence of the cyclic hexapeptide (CFWKYC) which is the minimal core involved for the activity of these peptides [31] and which is also present in UII and URP [27,28]. Previous docking [66,67] and photolabelling studies [68] showed that the side chains of Phe^6^ and Lys^9^ of *h*UII interact respectively with the Met^185^ residue located in the fourth transmembrane domain (TMD) and the Asn^130^ residue in the third TMD of the *h*UII receptor. The lower potencies of URP1 and URP2 toward *h*UT compared to *h*UII and *m*URP, the natural cognate peptides of this receptor, might be attributed to their longer C-terminus end. Indeed, steric hindrance and/or chemical nature of these three residues (VTN and SQN for URP1 and URP2 respectively) could interfere with the optimal positioning of the peptides within their putative hydrophobic binding pocket or their interaction with the second and third extracellular loop of the human receptor as shown for UII and URP [69].

UT has long been considered to be the only high affinity receptor for the UII family peptides, at least in mammals [7]. However, in a recent study, Tostivint et al. [28] provided evidence that the vertebrate ancestor likely possessed five distinct UT subtype genes, called UTS2R1–5 and that most of them have been preserved in teleosts. In zebrafish for example, four UT-like sequences have been identified that correspond to Uts2r1, the UT homologue, Uts2r2, uts2r3 and Uts2r4, while in stickleback, Uts2r2 seems to have been lost but is replaced by Uts2r5 [28]. In contrast, only the UTS2R1 subtype is still present in mammals. Considering the large number of UT receptor subtypes in teleosts, it is highly probable that URP1 and URP2 are able to bind to more than one receptor subtype. In support with this view, it is noteworthy that the Met^185^ and Asn^130^ residues mentioned above are conserved in the four putative UII receptors identified in zebrafish (data not shown). It is now evident that further studies will be needed to determine which receptor subtype the different UII-related peptides can preferentially bind to. In this regard, it will be interesting to determine whether the Uts2r1 subtype, the *h*UT counterpart studied in the present work, is the most efficient target of URP1 and URP2 or not.

In conclusion, we show here that, in the spinal cord, *urp1* and *urp2* are colocalized mainly in CSF-cNs, while, in the hindbrain, *urp1* but not *urp2* is confined to motoneurons in the glossopharyngeal-vagal motor nerve nuclei. We also demonstrate that URP1 and URP2 are active peptides toward human urotensin II receptor with similar potencies. Taken together, the functional redundancy of URP1 and URP2 as well as the colocalization of their mRNAs in the spinal cord suggest the robustness of this peptidic system and its physiological importance in zebrafish. These results provide the basis for further studies to improve our understanding of the physiological functions of URP1 and URP2 by using zebrafish as an experimental model.

## Supporting Information

S1 Fig *urp2* mRNA is found in cells contacting the fourth ventricle in the zebrafish adult brain. (TIF)

S2 Fig *urp1* ^+^ cells in the hindbrain do not express *gad*_*67*_ in adult zebrafish. (TIF)

S1 Table Sequences of the primers used for PCR amplifications (DOCX)
