# Supplementary figures and images for: Comparative Distribution and In Vitro Activities of the Urotensin II-Related Peptides URP1 and URP2 in Zebrafish: Evidence for Their Colocalization in Spinal Cerebrospinal Fluid-Contacting Neurons
Source: PLoS One. 2015 Mar 17;10(3):e0119290. doi: 10.1371/journal.pone.0119290 (PMC4364556; doi:10.1371/journal.pone.0119290)

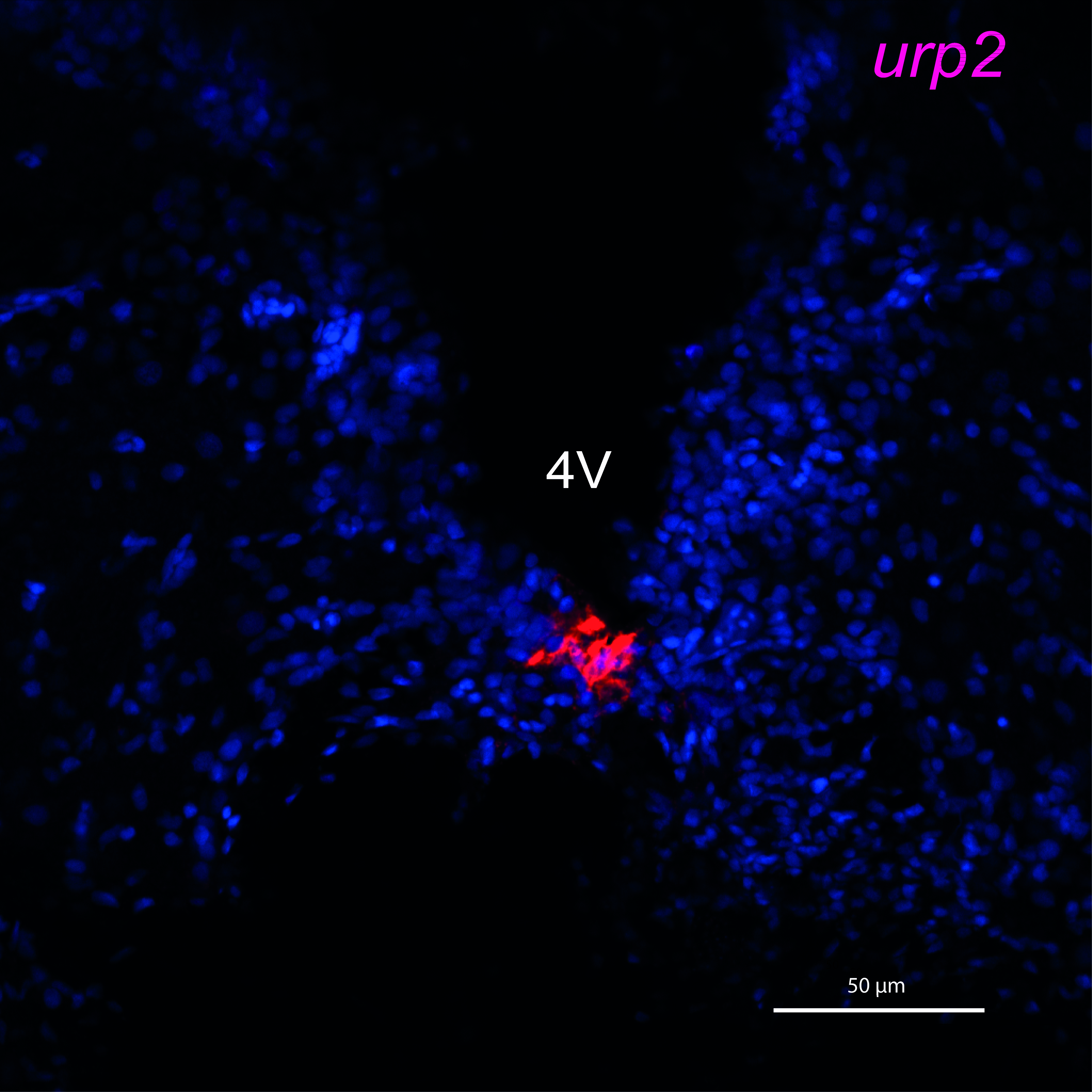

Supplement: S1 Fig — (TIF) [file pone.0119290.s001.tif]

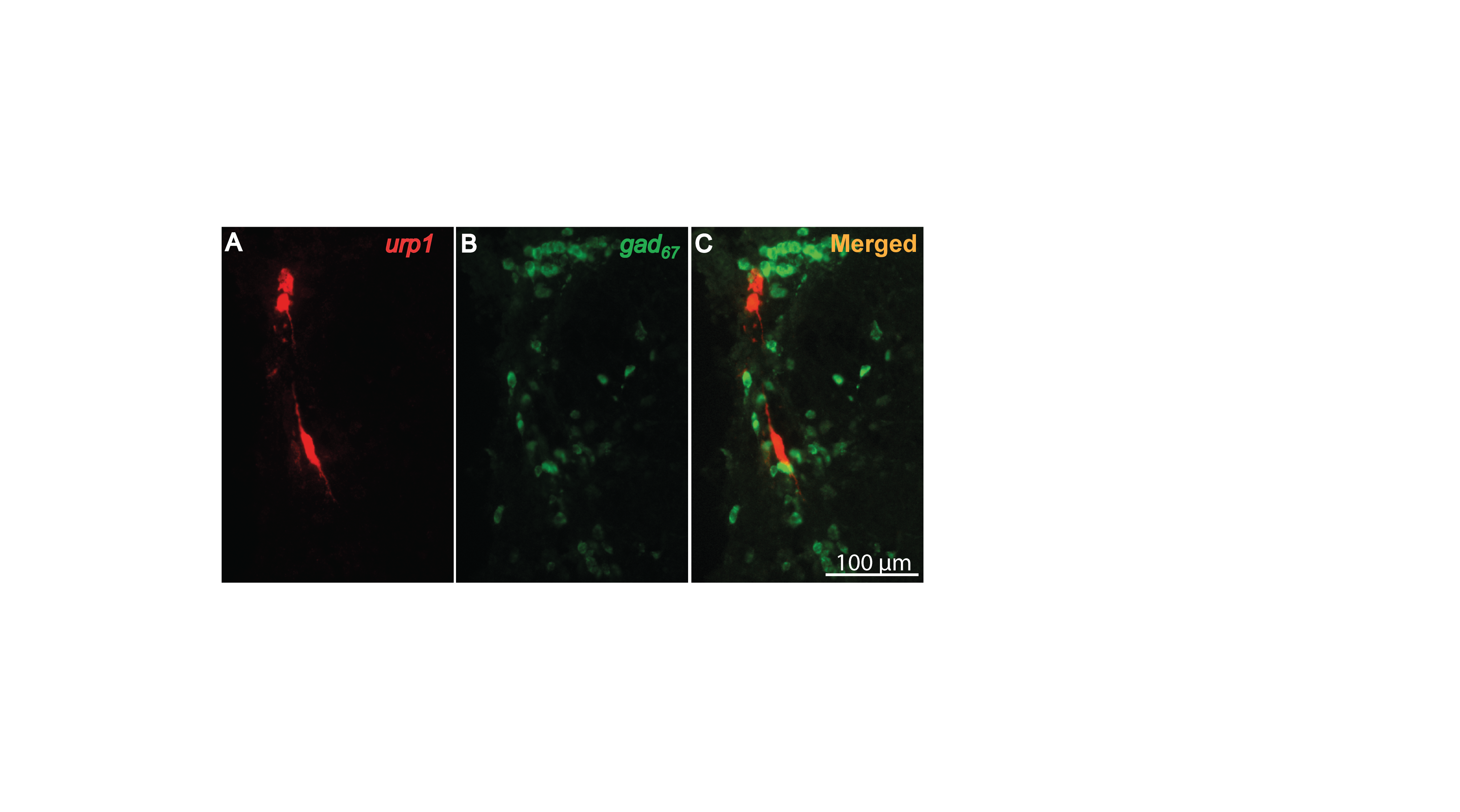

Supplement: S2 Fig — (TIF) [file pone.0119290.s002.tif]
