# Supplementary material for: Comparative Distribution and In Vitro Activities of the Urotensin II-Related Peptides URP1 and URP2 in Zebrafish: Evidence for Their Colocalization in Spinal Cerebrospinal Fluid-Contacting Neurons
Source: PLoS One. 2015 Mar 17;10(3):e0119290. doi: 10.1371/journal.pone.0119290 (PMC4364556; doi:10.1371/journal.pone.0119290)
Supplement: S1 Table — (DOCX) [file pone.0119290.s003.docx]

| Tissue distribution analysis | | |
| --- | --- | --- |
| URP1 | URP1For | TCTGGCTGTGGTTTGTTCTG |
|  | URP1Rev | CACAATGTAAAAGGGCAGCA |
| URP2 | URP2 For | AGACAGCGGTGGCATCTCA |
|  | URP2 Rev | TGGAGCTTGCAATAAGGAAGA |
| β-Actine | β-Act For | TCACCACCACAGCCGAAAG |
|  | β-Act Rev | GGTCAGCAATGCCAGGGTA |
| Probe synthesis | | |
| URP1 | URP1For | TCTGGCTGTGGTTTGTTCTG |
|  | URP1Rev | CACAATGTAAAAGGGCAGCA |
| URP2 | URP2’ For | TTGACCACAGTTGCTCTGCT |
|  | URP2 Rev | TGGAGCTTGCAATAAGGAAGA |
